# Tumour suppressor function of MDA-7/IL-24 in human breast cancer

**DOI:** 10.1186/1475-2867-10-29

**Published:** 2010-08-24

**Authors:** Neill Patani, Anthony Douglas-Jones, Robert Mansel, Wen Jiang, Kefah Mokbel

**Affiliations:** 1 Department of Breast Surgery, The London Breast Institute, The Princess Grace Hospital, 42-52 Nottingham Place, W1U-5NY, London, UK; 2 Department of Medical Genetics, Haematology & Pathology, Cardiff University School of Medicine, Cardiff University, Heath Park, CF14-4XN, Cardiff, UK; 3 Department of Surgery, Anaesthetics, Obstetrics & Gynaecology, Cardiff University School of Medicine, Cardiff University, Heath Park, CF14-4XN, Cardiff, UK; 4 Metastasis & Angiogenesis Research Group, University Department of Surgery, Cardiff University School of Medicine, Cardiff University, Heath Park, CF14-4XN, Cardiff, UK; 5 Department of Breast Surgery, St George's, University of London, Cranmer Terrace, SW17-0RE, London, UK; 6 Department of Biosciences, School of Health Sciences and Social Care, Brunel Institute of Cancer Genetics and Pharmacogenomics, Brunel University, Uxbridge, Middlesex, UB8 3PH, London, UK

## Abstract

**Introduction:**

Melanoma differentiation associated gene-7 (MDA-7), also known as interleukin (IL)-24, is a tumour suppressor gene associated with differentiation, growth and apoptosis. However, the mechanisms underlying its anti-neoplastic activity, tumour-specificity and efficacy across a spectrum of human cancers have yet to be fully elucidated. In this study, the biological impact of MDA-7 on the behavior of breast cancer (BC) cells is evaluated. Furthermore, mRNA expression of MDA-7 is assessed in a cohort of women with BC and correlated with established pathological parameters and clinical outcome.

**Methods:**

The human BC cell line MDA MB-231 was used to evaluate the in-vitro impact of recombinant human (rh)-MDA-7 on cell growth and motility, using a growth assay, wounding assay and electric cell impedance sensing (ECIS). Localisation of MDA-7 in mammary tissues was assessed with standard immuno-histochemical methodology. BC tissues (n = 127) and normal tissues (n = 33) underwent RNA extraction and reverse transcription, MDA-7 transcript levels were determined using real-time quantitative PCR. Transcript levels were analyzed against tumour size, grade, oestrogen receptor (ER) status, nodal involvement, TNM stage, Nottingham Prognostic Index (NPI) and clinical outcome over a 10 year follow-up period.

**Results:**

Exposure to rh-MDA-7 significantly reduced wound closure rates for human BC cells in-vitro. The ECIS model demonstrated a significantly reduced motility and migration following rh-MDA-7 treatment (p = 0.024). Exposure to rh-MDA-7 was only found to exert a marginal effect on growth. Immuno-histochemical staining of human breast tissues revealed substantially greater MDA-7 positivity in normal compared to cancer cells. Significantly lower MDA-7 transcript levels were identified in those predicted to have a poorer prognosis by the NPI (p = 0.049) and those with node positive tumours. Significantly lower expression was also noted in tumours from patients who died of BC compared to those who remained disease free (p = 0.035). Low levels of MDA-7 were significantly correlated with a shorter disease free survival (mean = 121.7 vs. 140.4 months, p = 0.0287) on Kaplan-Meier survival analysis.

**Conclusion:**

MDA-7 significantly inhibits the motility and migration of human BC cells in-vitro. MDA-7 expression is substantially reduced in malignant breast tissue and low transcript levels are significantly associated with unfavourable pathological parameters, including nodal positivity; and adverse clinical outcomes including poor prognosis and shorter disease free survival. MDA-7 offers utility as a prognostic marker and potential for future therapeutic strategies.

## Introduction & Background

Melanoma differentiation associated gene-7 (MDA-7), also known as interleukin (IL)-24, is an intriguing member of the class II/IL-10 cytokine family [1]. This novel tumour suppressor gene was initially identified from human melanoma cells [2,3]. Mapped within the IL-10 family cytokine cluster to 1q32.2-q41, the gene encodes a protein consisting of 206 amino acids, secreted in mature form as a 35-40 kDa phosphorylated glycoprotein [4,5]. MDA-7 is expressed by diverse cell types, including: B cells, Nk cells, dendritic cells, monocytes and melanocytes. Although its physiological role is poorly understood, forced expression of MDA-7 in cancer cells results in irreversible growth inhibition, reversal of the malignant phenotype and terminal differentiation [6]. Further in-vitro and in-vivo studies have demonstrated these attributes to be tumour-selective and applicable to numerous solid malignancies. Many human cancer derived cell lines, including: prostate, breast, cervical, lung, fibrosarcoma, colorectal, melanoma, and glioblastoma, undergo apoptosis when exposed to MDA-7 [7-12]. Interestingly, similar effects are not apparent following transduction into their non-malignant counterparts [13]. Specific anti-tumour activity has also been established in a range of human tumour xenograft models and recently in several early-phase clinical trials involving patients with advanced solid cancers [10,11,14,15]. MDA-7 is emerging as a differentiation, growth and apoptosis associated gene with potential utility for the gene-based therapy of several human cancers [4].

However, the mechanisms through which MDA-7 expression exerts its anti-neoplastic activity, tumour-specificity and efficacy across a spectrum of human cancers have yet to be fully elucidated. Akin to other cytokines, secreted MDA-7 operates via its cell surface receptor complex, involving the IL-20R1/IL-20R2 or IL-22R1/IL20R2 hetero-dimers [16,17]. Although receptor activation is associated with the Janus activated kinase (JAK)/signal transducers and activators of transcription (STAT) signalling, specific tumour suppressor function may not be entirely dependent upon these pathways [18]. Indeed, selective anti-tumour activity is believed to be exerted through both secretory (extra-cellular) and non-secretory (intra-cellular) pathways [19,20]. Intra-cellular activity is thought to be mediated in a cell surface receptor-independent manner, involving endoplasmic reticulum (ER) signalling, as evidenced by localisation studies, interaction with resident ER chaperones and the induction of particular genes associated with the ER stress response [13,21-23]

In this study, the biological impact of MDA-7 on the behavior of breast cancer (BC) cells is evaluated. Furthermore, mRNA expression of MDA-7 is assessed in a cohort of women with BC. Transcript levels are compared with normal background tissues and evaluated against established pathological parameters and clinical outcome over a 10 year follow-up period.

## Methods

### Patients, materials and cell lines

The human BC cell line MDA MB-231 was obtained from ATCC (American Type Cell Collection, Maryland, USA). BC tissues (n = 127) and normal background tissues (n = 31) were collected from University Hospital of Wales and St George's Hospital and Medical School; institutional guidelines, including ethical approval and informed consent, were followed. Specimens were obtained immediately after excision during surgery and stored at -80°C until use. A consultant pathologist examined haematoxylin and eosin stained frozen sections to verify the presence of tumour cells in the collected samples. Normal tissue was derived from the background breast parenchyma of BC patients within the study group. Medical notes and histology reports were used to extract the clinico-pathological data (Table 1). A customized database was established to record the data. Recombinant human (rh) MDA-7/IL-24 was purchased from R&D System Europe. Antibodies to human MDA-7/IL-24, anti-IL20Rα, anti-IL20Rβ, and anti-IL-22R were obtained from Santa-Cruz Biotechnologies Inc. ROCK inhibitor was purchased from Santa-Cruz Biotechnologies Inc., Akt inhibitor, SIS3 inhibitor, PLC-gamma inhibitor, JNK inhibitor, JAK inhibitor, MET inhibitor, Wortmannin and Wiskostatin were obtained from Calbiochem, Nottingham, England, UK. Matrigel (reconstituted basement membrane) was purchased from Collaborative Research Products (Bedford, Massachusetts, USA). Transwell plates equipped with a porous insert (pore size 8 μm) were obtained from Becton Dickinson Labware (Oxford, UK). DNA gel extraction and plasmid extraction kits were purchased from Sigma.

**Table 1:** Clinical and pathological data

Parameter	Category	Number
**Node Status**	Node positive	54
	Node negative	73
**Tumour Grade**	1	24
	2	43
	3	58
**Tumour Type**	Ductal	98
	Lobular	14
	Medullary	2
	Tubular	2
	Mucinous	4
	Non specific	7
**TNM staging**	1	70
	2	40
	3	7
	4	4
**NPI**	NPI1	68
	NPI2	38
	NPI3	16
**Clinical Outcome**	Disease free	90
	Alive with metastasis	7
	With local recurrence	5
	Died from breast cancer	16
	Died of unrelated disease	9
**ER status**	ER α negative	75
	ER α positive	38
	ER β negative	91
	ER β positive	24

Note: missing values reflect discarded/un-interpretable values

### Tissue processing, RNA extraction, cDNA synthesis and RT-PCR

Frozen sections of tissue were cut at a thickness of 5-10 mm and kept for routine histological analysis. Additional 15-20 sections were mixed and homogenized using a hand-held homogenizer in ice-cold RNA extraction solution. RNA from cells was extracted using an RNA extraction kit (AbGene Ltd, Surrey, England, UK). RNA concentration was quantified using a UV spectrophotometer (Wolf Laboratories, York, England, UK). Reverse transcription was carried out using a reverse transcription kit, cDNA was synthesised using first strand synthesis with an anchored oligo^dt ^primer (AbGene, Surrey, UK). The polymerase chain reaction (PCR) was performed using sets of primers (Table 2) with the following conditions: 5 min at 95°C, 20 seconds at 94°C, 25 seconds at 56°C, 50 seconds at 72°C for 36 cycles and finally 72°C for 7 minutes. β-actin was amplified and used as a house keeping control to verify the quality of cDNA. PCR products were separated on a 0.8% agarose gel, visualised under UV light, photographed using a Unisave™ camera (Wolf Laboratories, York, England, UK) and recorded with Photoshop software.

**Table 2:** Forward and reverse primers

MDA-7/IL-24 F	GATGTTTTCCATCAGAGACAG
MDA-7/IL-24 Zr	ACTGAACCTGACCGTACACATCCAGGTCAGAAGAATGT
IL20R1 F	TACAATGGACTCCACCAGAG
IL20R1 ZR	ACTGAACCTGACCGTACATATTCAGCCATTTCTTTTGC
IL20R2 F	GCCTGGAGAAACAGTGTACTA
IL20R2 ZR	ACTGAACCTGACCGTACACAGGACCTTCAGTGAGTGAG
CK-19 F	CAGGTCCTAGAGGTTACTGAC
CK-19 Zr	ACTGAACCTGACCGTACACACTTTCTGCCAGTGTGTCTTC
β-actin F	ATGATATCGCCGCGCTCGTC
β-actin Zr	CGCTCGGTGAGGATCTTCA

### Quantitative analysis of MDA-7

MDA-7 transcript levels within the above-prepared cDNA was determined using real-time quantitative PCR, based on the Amplifluor™ technology, modified from previous reports [24,25]. Pairs of PCR primers were designed using the Beacon Designer™ software (Version 2, Palo Alto, California, USA) and synthesized by Sigma-Aldrich, added to the reverse primer was an additional sequence, known as the Z sequence (5'-ACTGAACCTGACCGTACA-'3) which is complementary to the universal Z probe (Intergen Inc., Oxford, England, UK). The product expands one intron. The primers used are detailed in Table 2. Taqman detection kit for β-actin was purchased from Perkin-Elmer. The reaction was carried out using the following: custom made hot-start Q-master mix Abgene (Surrey, England, UK), 10 pmol of specific forward primer, 1 pmol reverse primer with the Z sequence (Table 2), 10 pmol of FAM- (fluorogenic reporter dye, carboxyfluorescein) tagged probe (Intergen Inc.), and cDNA generated from 50 ng RNA. The reaction was carried out using IcyclerIQ™ (Bio-Rad, Hemel Hempstead, England, UK) which is equipped with an optic unit that allows real-time detection of 96 reactions, under the following conditions: 94°C for 12 minutes, 50 cycles of 94°C for 15 seconds, 55°C for 40 seconds and 72°C for 20 seconds. The transcript levels were generated from an internal standard that was simultaneously amplified with the samples. The levels of gene expression were then normalized against the housekeeping control, which was also quantified in these specimens, to correct for varying amounts of epithelial tissue between samples [26]. With every PCR run, a negative control without a template and a known cDNA reference sample as a positive control, were included.

### In-vitro cell growth assay

Cells were plated into 96-well plates at 2,000 cells/well, followed by a period of incubation. Cells were fixed in 10% formaldehyde at the day of plating and daily for the subsequent 5 days. 0.5% crystal violet (w/v) was used to stain cells. Following washing, the stained crystal violet was dissolved with 10% (v/v) acetic acid and the absorbance was determined at a wavelength of 540 nm using an ELx800 spectrophotometer. Absorbance was used to represent the cell number.

### Electric Cell-substrate Impedance Sensing (ECIS) based cell adhesion assay

Cell migration was determined using a wounding assay and ECIS assays. Two models of ECIS instrument were used: ECIS 9600 for screening and ECIS1600R for modelling. In both systems, 8W10 arrays were used (Applied Biophysics Inc, NJ, US). The array surfaces and electrodes were pre-treated with a Cysteine solution (10 mM) and subsequently incubated with complete medium for an hour. The same number of BC cells was added to each well (300,000/well). Electric changes were continuously monitored for up to 24 hours. In the 9600 system, the monitoring was at fixed 30 Hz. In the 1600R system, two conditions were recorded: 400 Hz, 4,000 Hz, 40,000 Hz for screening the cells response to IL-24/MDA-7 and 4,000 Hz fix frequency for cell modelling.

### Immuno-histochemical analysis of MDA-7 & MDA-7 receptors in cells and tissues

Frozen sections of breast tissues (normal and tumour) were cut at a thickness of 6 μm using a cryostat. Sections were mounted on super frost plus microscope slides, air dried and then fixed in a mixture of 50% Acetone and 50% methanol. Sections were then placed in "Optimax" wash buffer for 5-10 minutes to rehydrate. Sections were incubated for 20 minutes in a 0.6% bovine serum albumin (BSA) blocking solution and probed with the primary antibody. Sections were incubated for 20 minutes in a 10% horse serum blocking solution and probed with the primary antibodies (1:100 for anti-MDA-7, anti-IL-20Rα, and 1:150 for anti-IL20Rβ and anti-IL22R). Following extensive washings, sections were incubated for 30 minutes in the secondary biotinylated antibody (Multi-link Swine anti-goat/mouse/rabbit immunoglobulin, Dako Inc.). Following washings, Avidin Biotin Complex (Vector Laboratories) was then applied to the sections followed by further washings. Di-amino-benzidine (DAB) chromogen (Vector Labs) was then added to the sections which were incubated in the dark for 5 minutes. Sections were counter stained with Gill's Haematoxylin and dehydrated in ascending grades of methanol before clearing in xylene and mounting under a cover slip.

### Statistical analysis

The two-sample *t*-test (comparison of mean copy number) was used for statistical analysis of absolute and normalised gene copy number. For normality the Anderson-Darling test was used. The transcript levels within the BC specimens were compared to normal background tissues and analyzed against conventional pathological parameters and clinical outcome over a 10 year follow-up period. In each case the true copy number was used for statistical analysis and hence the samples were not classified as positive or negative. The statistical analysis was carried out using Minitab version 14.1 (Minitab Ltd. Coventry, England, U.K.) using a custom written macro (Stat 2005.mtw). For purposes of the Kaplan-Meier survival analysis, the samples were divided arbitrarily into two groups, 'high transcript level' or 'low transcript level'. The cut-off was guided by the Nottingham Prognostic Index (NPI) value, with which the value of the moderate prognostic group was used as the dividing line at the start of the test. Survival analysis was performed using SPSS version 16.0 (SPSS Inc. Chicago, IL, USA). NPI = tumour size (cm) × 0.2 + lymph node stage (1 - no nodes affected; 2 - up to 3 nodes affected; 3 - more than 3 nodes affected) + Grade (1-3, Scarff-Bloom-Richardson). NPI scores were classified into three groups: < 3.4 = NPI-1, 3.4-5.4 = NPI-2, > 5.4 = NPI-3. Within tumour samples, oestrogen receptor (ER) status was classified according to transcript copy number per 50 ng (nanograms) of RNA: < 1 = negative, ≥1 = positive.

## Results

**Figure 1:**
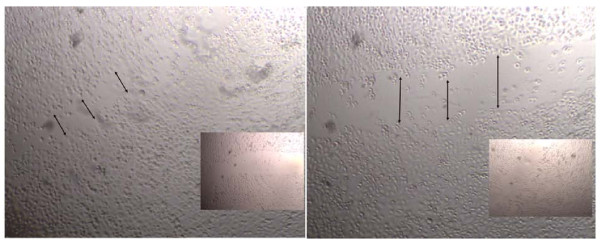
**The impact of rh-MDA-7/IL-24 on BC cell migration**. Exposure to rh-MDA-7 significantly reduced the wound closure rate of MDA MB-231 cells, after scratch wounding. Left: control; Right: Cells treated with rh-MDA-7 at 20 ng/ml. Photographs shown are 2 hours after wounding. Inserts: beginning of the wounding at lower magnification.

**Figure 2:**
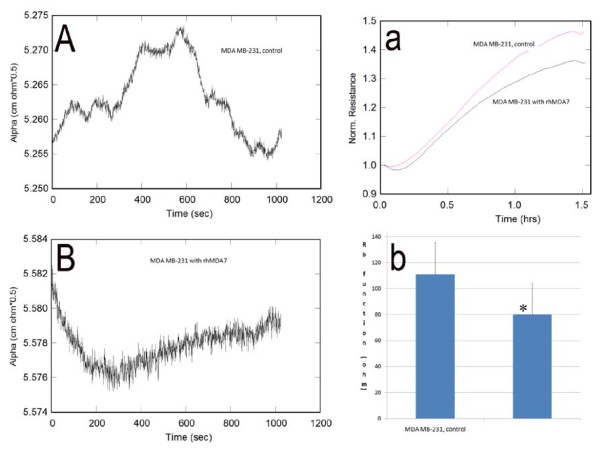
**Impact of rh-MDA-7 on the micro-motion (A, B) and migration (a, b) of the BC cell line MDA MB-231, using the ECIS model (1600R for A, B and 9600 for a, b)**. The presence of rh-MDA-7 significantly reduced the motility of BC cells.

The MDA MB 231 cell line was confirmed to express both IL20R1 (0.008 copies/ul) and IL-20R2 (40.5 copies/ul). In-vitro studies demonstrated that migration of BC cells was profoundly affected by rh-MDA-7. After scratch wounding, exposure to rh-MDA-7 significantly reduced the wound closure rate of MDA MB-231 cells treated with rh-MDA-7 at 20 ng/ml, when compared to controls. The impact of rh-MDA-7/IL-24 on BC cell migration is depicted in Figure 1. The ECIS model confirmed that MDA MB-231 cells treated with rh-MDA-7 showed a significantly slower rate of migration (electrical resistance 80.1 ± 24.3), when compared with control cells (130 ± 24.3), p = 0.024. Furthermore, the presence of rh-MDA-7 significantly reduced the motility of BC cells. The impact of rh-MDA-7 on the micro-motion and migration of the MDA MB-231 cell line is depicted in Figure 2. Exposure to rh-MDA-7 was only found to exert a marginal effect on in-vitro growth which did not reach statistical significance.

**Figure 3:**
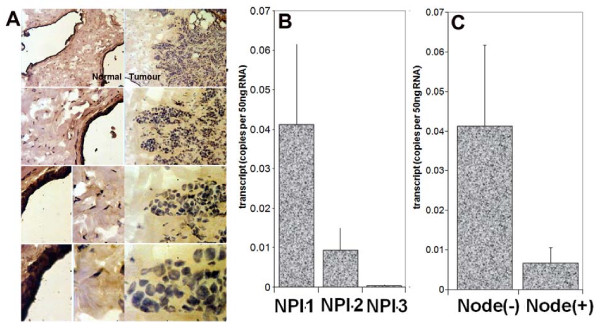
**Localisation of MDA-7 in mammary tissues (A) and levels of MDA-7 transcript in breast cancer tissues in relation to prognosis (B) and nodal status (C)**. Breast cancer cells stained virtually negative for MDA-7, in comparison with normal epithelial cells (A-left). Tumours from patients predicted to have a poorer prognosis and node positive tumours had lower transcript levels (B and C respectively).

**Figure 4:**
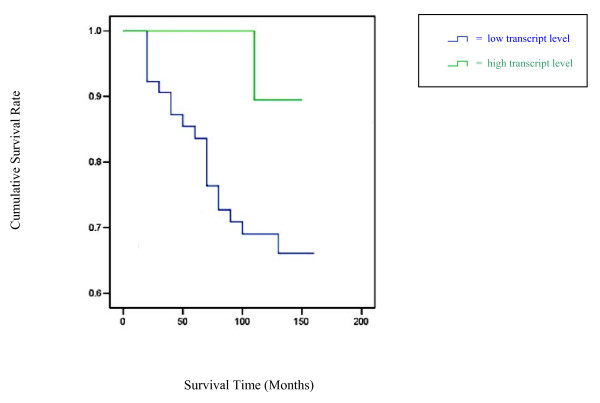
**MDA-7 expression and Kaplan-Meier survival analysis**. Low levels of MDA-7 are significantly correlated with a shorter disease free survival (mean = 121.7 months, 95% CI = 108.5-134.9) compared with high levels (mean = 140.4 months, 95% CI = 133.7 - 147.1), p = 0.0287.

Immuno-histochemical staining of human breast tissues revealed a substantially greater degree of MDA-7 positivity within normal mammary epithelial cells, compared to virtually no staining in cancer cells, Figure 3A. Using the NPI as a prognostic indicator, tumours from patients with poorer prognosis showed significantly lower MDA-7 transcript levels compared with their counterparts predicted to have a good prognosis (p = 0.049), Figure 3B. MDA-7 transcript levels were also found to be correlated with nodal status, with lower transcript levels found in node positive tumours, Figure 3C. Patients who remained alive and disease free had a significantly higher levels of MDA-7 compared with those who developed distant metastasis (3.77 ± 1.32 vs. 0.0019 ± 0.002, p = 0.0058). A significant difference in MDA-7 expression was identified between tumours from patients who died of breast cancer and those who remained disease free after a median follow up of 10 years, with the latter showing higher MDA-7 transcript levels, p = 0.035. Furthermore, Kaplan-Meier survival analysis revealed that low levels of MDA-7 were significantly correlated with a shorter disease free survival (mean = 121.7 months, 95% CI = 108.5-134.9) compared with high levels of expression (mean = 140.4 months, 95% CI = 133.7-147.1), p = 0.0287, Figure 4. The MDA-7/CK-19 ratio was also found to have significant predictive value for disease free survival (p = 0.0435). Lower MDA-7 transcript levels were associated with a shorter overall survival, although this did not reach statistical significance (p = 0.078 for MDA-7, p = 0.1435 for MDA-7/CK-19 ratio). ER positive tumours were found to have lower levels of MDA-7 expression compared with ER-negative tumours, although this trend did not reach statistical significance (p = 0.094, NS).

## Discussion

The present study adds to the literature in support of the tumour suppressor function of MDA-7 in solid human malignancies, with particular reference to BC. Furthermore, this study is the first to quantitatively evaluate MDA-7 mRNA expression in a large cohort of BC patients and provide correlation with conventional pathological parameters and clinical outcomes over an extended follow-up period. MDA-7 expression was found to be substantially reduced in malignant breast tissue and low transcript levels were significantly associated with unfavourable pathological parameters, including nodal positivity; and adverse clinical outcomes including poor prognosis and shorter disease free survival. Despite the inferences drawn, the mechanisms through which MDA-7 expression exerts its tumour-specificity, anti-neoplastic activity and efficacy across a range of human cancers have yet to be fully elucidated and will undoubtedly be necessary to optimise potential therapeutic applications.

Several in-vitro studies, including the current study, have employed rh-MDA-7 for exposure testing in various experiments, however MDA-7 delivery can also be achieved via a non-replicating adenovirus, referred to as adenoviral-mediated gene transfer (Ad-MDA-7) or adenoviral-induced expression. Ni et al. [27] have also investigated the effects of MDA-7 on the growth and apoptosis of human BC cell lines. In contrast to the results of the present study which found MDA-7 to have only a marginal impact on tumour cell growth, their in-vitro transfection studies demonstrated a clear dose and time-dependent inhibition of cell growth in addition to enhanced apoptosis [27]. It is possible that differences in the method of MDA-7 delivery, in-vitro assay and cell lines employed could explain some of the discrepancies in efficacy noted between studies. MDA-7 is believed to be associated with the induction of key molecules, altering the balance between pro- and anti-apoptotic proteins which mediate growth inhibition and apoptosis in several tumour types. Mechanistic studies undertaken by Sauane et al. [5] have confirmed the specificity of MDA-7 for transformed cells and demonstrate localisation to the ER with the induction of sustained stress as evidenced by expression of ER stress markers. Apoptosis was found to be mediated through JAK/STAT independent and p38-mitogen-activated protein kinase (MAPK)-dependent pathways [5]. In keeping with this, Sarkar et al. [23] found that Ad-MDA-7 acts in a tumour-specific manner, resulting in the induction of various growth arrest and DNA damage inducible (GADD) genes, via p38-MAPK activation, associated with the stress response and ER stress pathways [5,23]. Gupta et al. [21] have also shown the importance of MDA-7 interaction with the ER resident chaperone BiP/GRP78 for induction of ER stress signals and subsequent activation of p38-MAPK and GADD gene expression which culminate in apoptosis. Lebedeva et al. [28] have demonstrated that these effects are not present in normal and non-malignant cells. Another interesting observation with mechanistic implications comes from Suh et al. [29], who have combined Ad-MDA-7 with a selective cyclo-oxygenase (COX)-2 inhibitor and identified synergistic in-vitro tumouricidal activity against BC cell lines. In addition to the aforementioned studies evaluating cell growth and survival, the present study demonstrated MDA-7 to have a profound inhibitory effect on the motility and migration of BC cells in-vitro. It would appear that MDA-7 has a regulatory role in cell motility with the ability to modulate and influence the pathways involved with implications for both local invasion and metastatic potential.

In-vivo, MDA-7 mediated tumour specific apoptosis has been demonstrated to be supplemented by an additional 'anti-tumour bystander' effect, with the former 'intra-cellular killing' being receptor-independent and the latter dependent on MDA-7 secretion and canonical IL-20/IL-22 receptor complexes on the surface of target cells [14,15,19,22]. In-vivo studies involving the inoculation of nude mice with human BC cell lines have demonstrated significant growth inhibition following MDA-7 injection [27]. Sarkar et al. [30] have devised a dual cancer-specific targeting strategy by constructing a conditionally replication competent adenovirus which demonstrates tumour-specific virus replication, in addition to the expression of MDA-7. Their results are encouraging, with the complete eradication of both primary and distant disease in human BC xenografts in nude mice [30]. MDA-7 has also been shown to influence endothelial cells, exerting an potentially anti-angiogenic effect within the tumour vasculature [31]. Chada et al. [32] found Ad-MDA-7 to mediate p53-independent inhibition of tumour growth, cell cycle arrest and apoptosis, associated with down-regulation of Bcl-2 and Akt. In-vivo, growth inhibition was demonstrated in multiple xenograft models. Furthermore, Ad-MDA-7 was demonstrated to have an additive or synergistic effect in both cellular and animal studies when combined with chemotherapy, biologic therapies and radiotherapy. These effects were associated with decreased Bcl-2 expression and BAX up-regulation [32]. Bocangel et al. [33] evaluated the treatment of a panel of Her-2/neu over-expressing cell lines and nude mice tumours with a combination of Ad-MDA-7 gene transfer and Trastuzumab/Herceptin, the anti-human epidermal growth factor receptor-2 (Her-2) monoclonal antibody. The study demonstrated synergistic tumour suppression with increased cell death, cell cycle block and apoptosis [33]. Their study is supported by that of McKenzie et al. [34].

Hence, in addition to utility as a prognostic marker, MDA-7 appears to offer significant therapeutic potential, enticing translational researchers with the prospect of tumour-specificity and efficacy against a range of solid human cancers. Indeed, therapeutic potential has been recently evaluated. The safety and efficacy of Ad-MDA-7 has been demonstrated in phase-1 clinical trials of intra-tumoural injection into several solid cancers, including melanoma [10,11,14,15]. Albeit premature, MDA-7 has already been referred to by some authors as the "magic bullet for cancer" and "cancer's Achilles' heel" [8,10]. However, further mechanistic studies are imperative to fully unlock and optimise future therapeutic applications.

Limitations of the present study include the single BC cell line evaluated and single method of MDA-7 delivery employed. In addition to motility, migration and growth, in-vitro studies could be extended to include assays of adhesion, invasiveness and apoptosis. Immuno-fluorescence could also be employed to evaluate changes in cyto-skeletal arrangement after treatment with rh-MDA-7. The use of background parenchyma from BC patients to provide 'normal tissue' for comparison is also contentious. Ideally, such material should be derived from patients without BC in order to avoid any 'field change' which may exist within cancer bearing tissues. Although the sample size and follow-up period were substantial, it is possible that a larger cohort, particularly with regard to subgroup analysis, may have influenced several results which approached, but failed to reach, statistical significance. In addition to the measurement of mRNA transcript levels, quantitative analysis of protein expression should be undertaken to ensure concordance. Correlation with associated molecules and other markers of invasiveness and metastatic competence would also have been of value.

## Conclusion

MDA-7 significantly inhibits the motility and migration of human BC cells in-vitro. MDA-7 expression is substantially reduced in malignant breast tissue and low transcript levels are significantly associated with unfavourable pathological parameters, including nodal positivity; and adverse clinical outcomes including poor prognosis and shorter disease free survival. In addition to its prognostic utility, further mechanistic studies are warranted to explore the potential for therapeutic manipulation in human breast cancer.

## Conflicts of interests

The authors declare that they have no competing interests.

## Authors' contributions

NP Literature Review, Data Interpretation, Manuscript Writing & Editing

ADJ Study Design/Concept, Pathological Overview

RM Study Design/Concept

WJ Study Design/Concept, Laboratory Methodology, Data Acquisition/Analysis

KM Principal Investigator, Study Design/Concept, Patient Recruitment, Data Analysis

All authors read and approved the final manuscript.
